# Supplementary figures and images for: Bioactive triterpenoids from Solanum torvum fruits with antifungal, resistance modulatory and anti-biofilm formation activities against fluconazole-resistant candida albicans strains
Source: PLoS One. 2021 Dec 28;16(12):e0260956. doi: 10.1371/journal.pone.0260956 (PMC8714089; doi:10.1371/journal.pone.0260956)

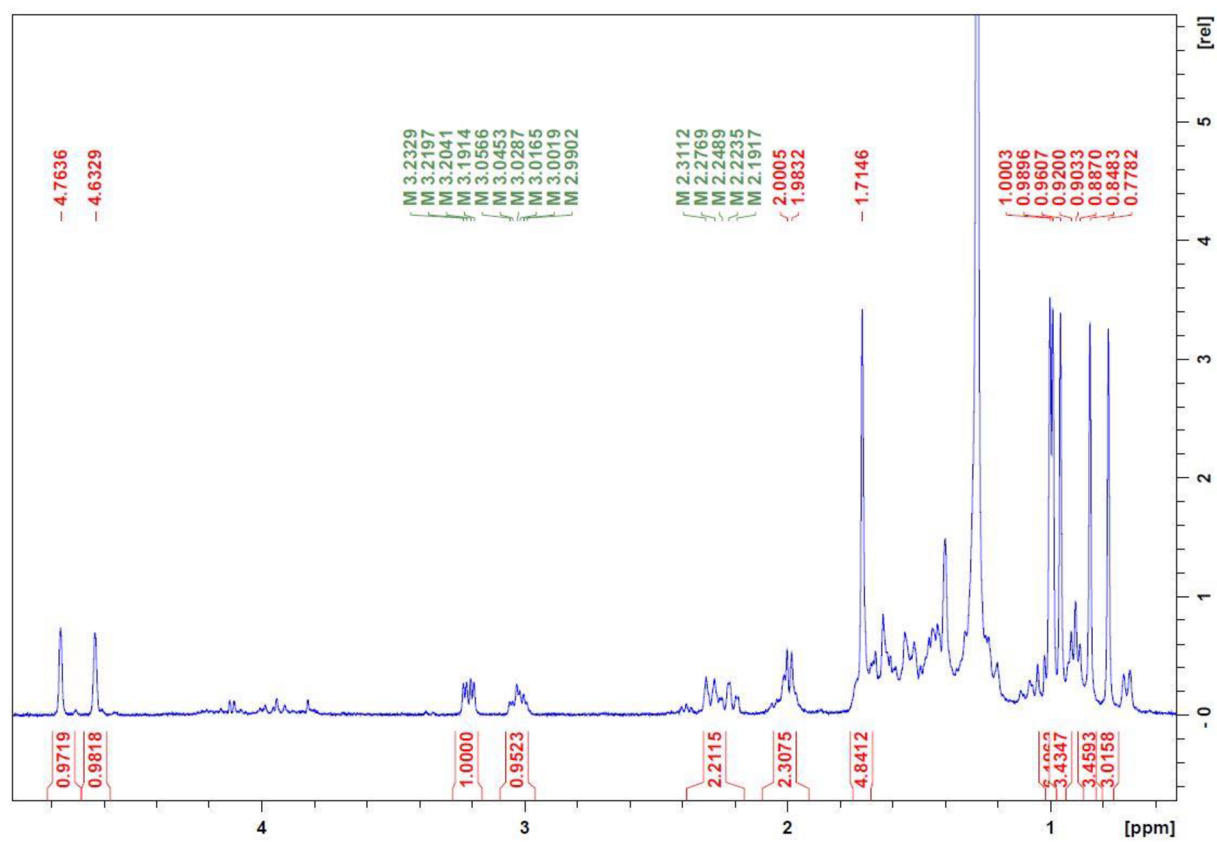

S1 Fig.  $^1\text{H}$  NMR spectrum of compound 1

Supplement: S1 Fig — (PDF) [file pone.0260956.s001.pdf]

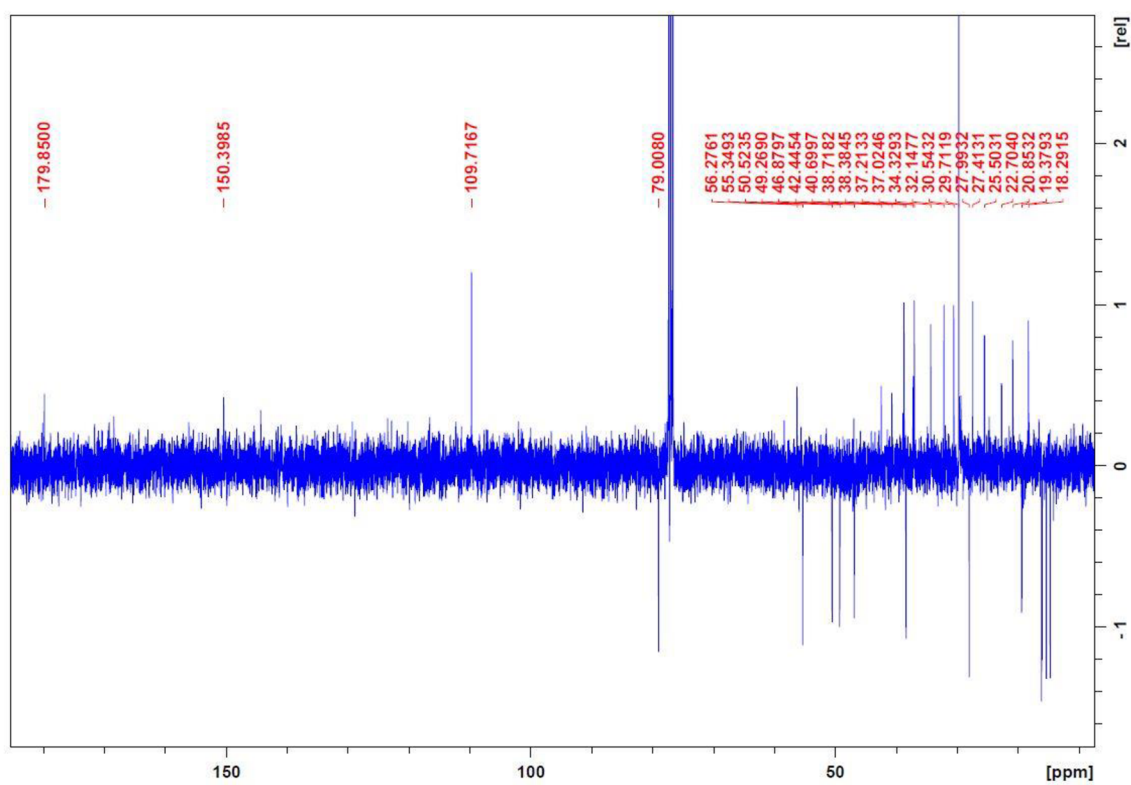

**S2 Fig. DEPT Q NMR spectrum of compound 1**

Supplement: S2 Fig — (PDF) [file pone.0260956.s002.pdf]

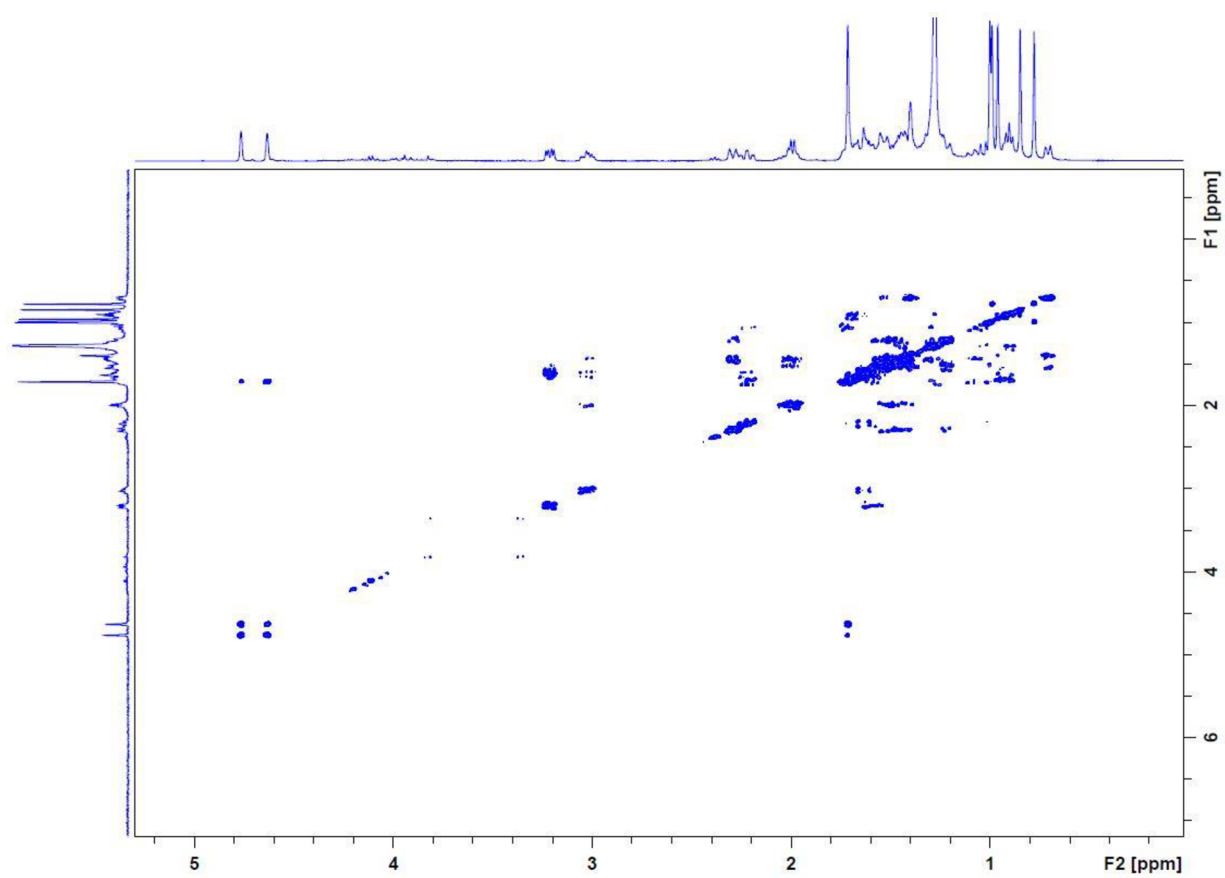

**S3 Fig. COSY spectrum of compound 1**

Supplement: S3 Fig — (PDF) [file pone.0260956.s003.pdf]

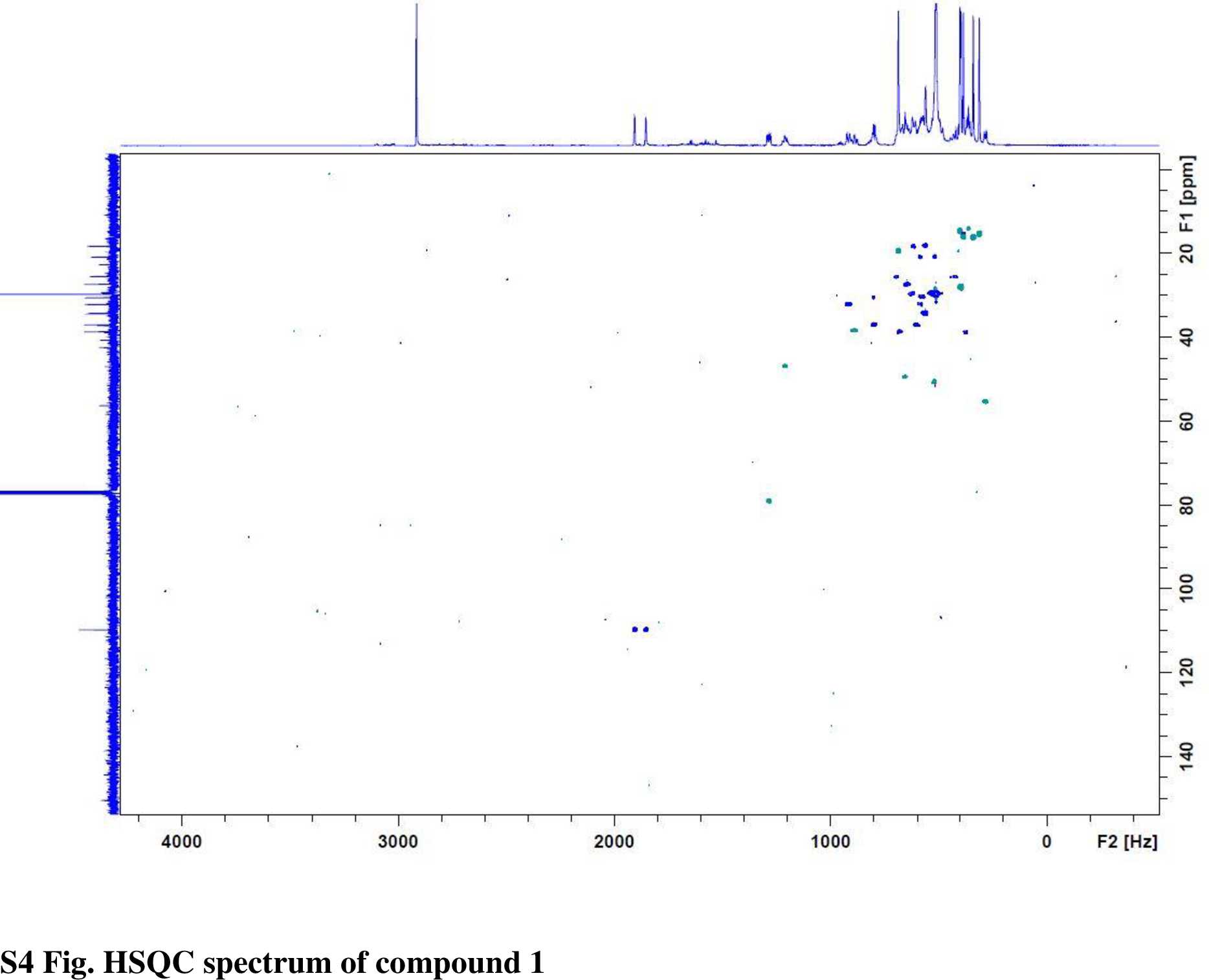

Supplement: S4 Fig — (JPG) [file pone.0260956.s004.jpg]

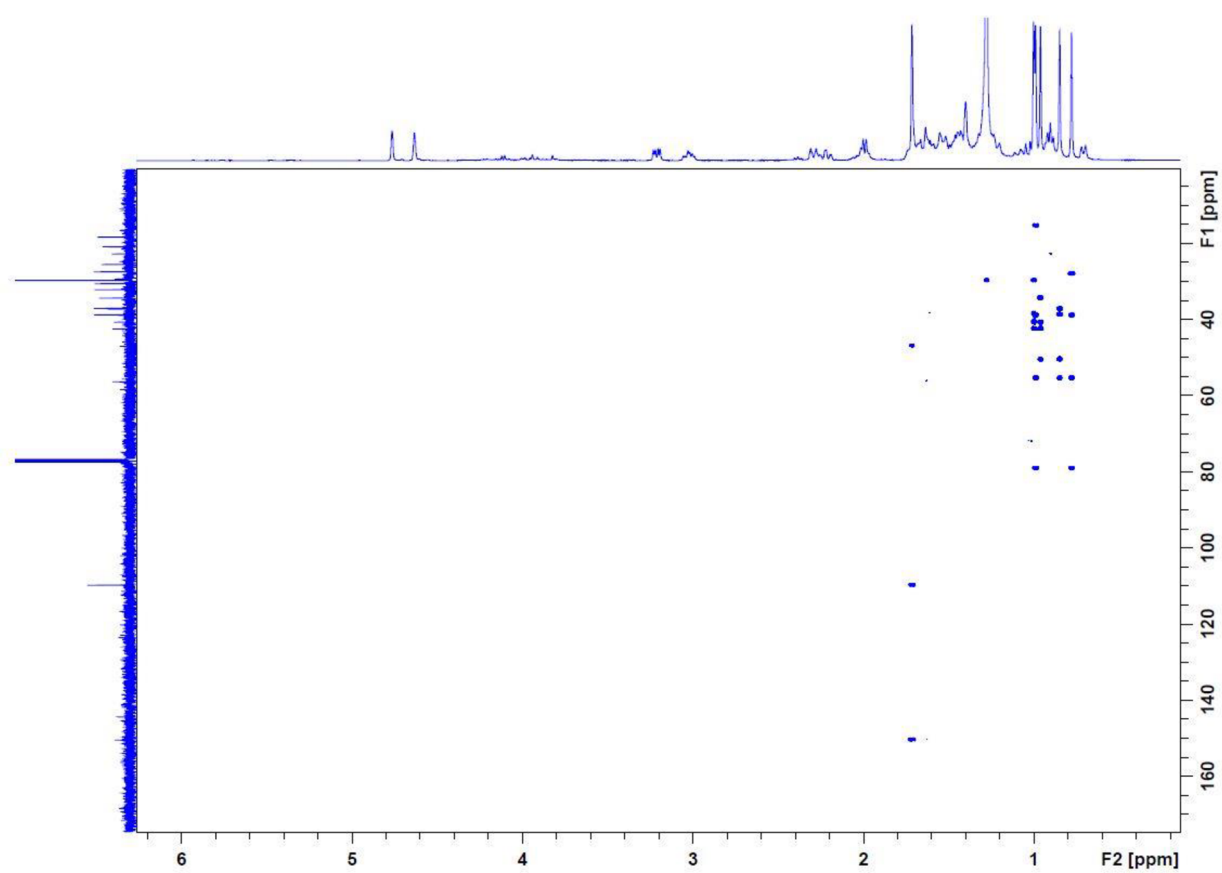

**S5 Fig. HMBC spectrum of compound 1**

Supplement: S5 Fig — (PDF) [file pone.0260956.s005.pdf]

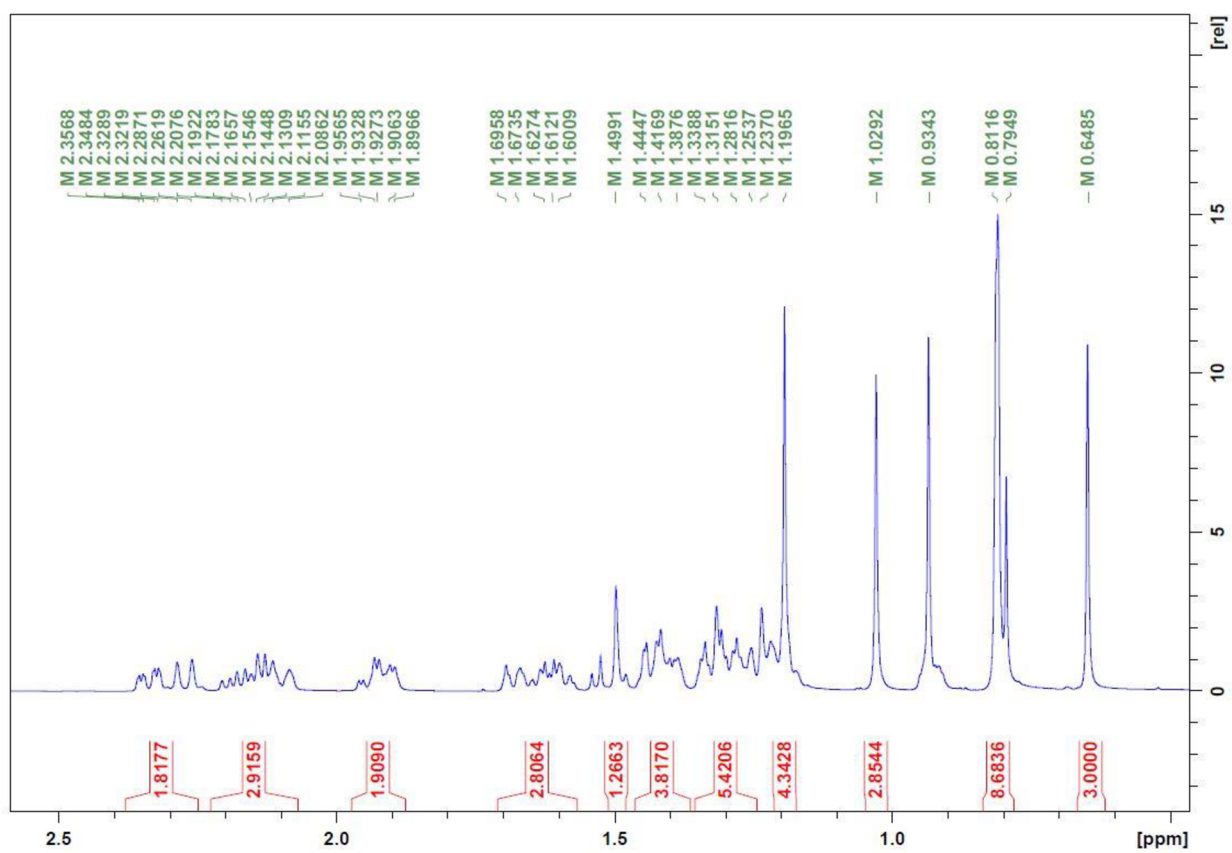

S6 Fig. <sup>1</sup>H NMR spectrum of compound 2

Supplement: S6 Fig — (PDF) [file pone.0260956.s006.pdf]

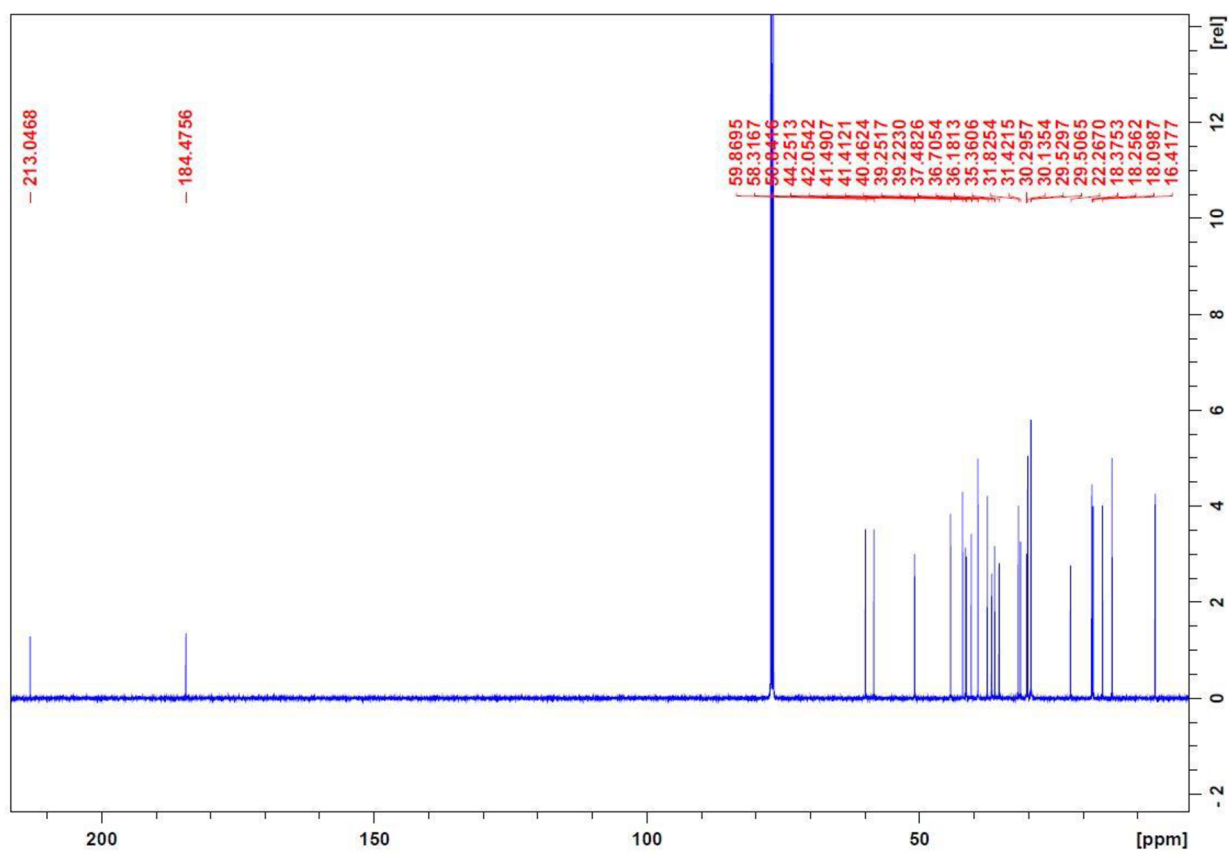

S7 Fig. <sup>13</sup>C NMR spectrum of compound 2

Supplement: S7 Fig — (PDF) [file pone.0260956.s007.pdf]

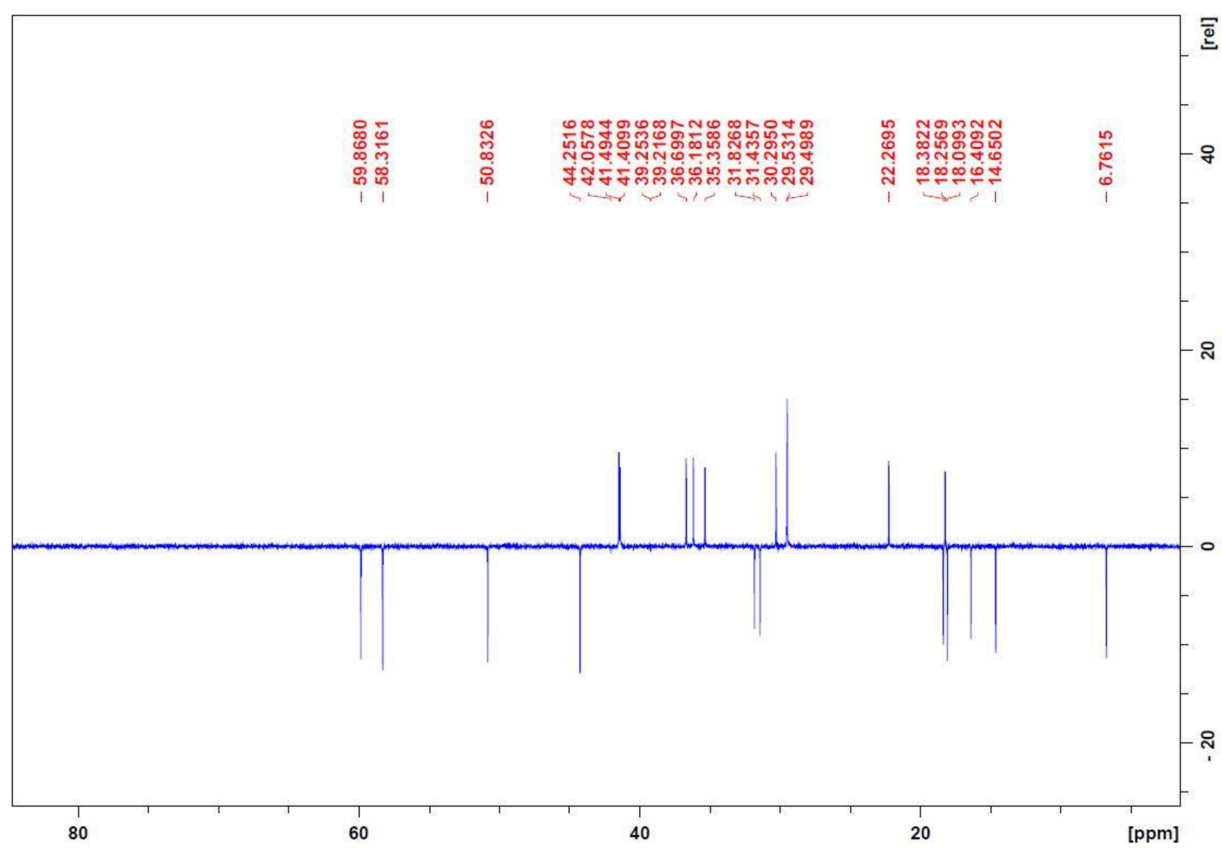

S8 Fig. DEPT 135 spectrum of compound 2

Supplement: S8 Fig — (PDF) [file pone.0260956.s008.pdf]

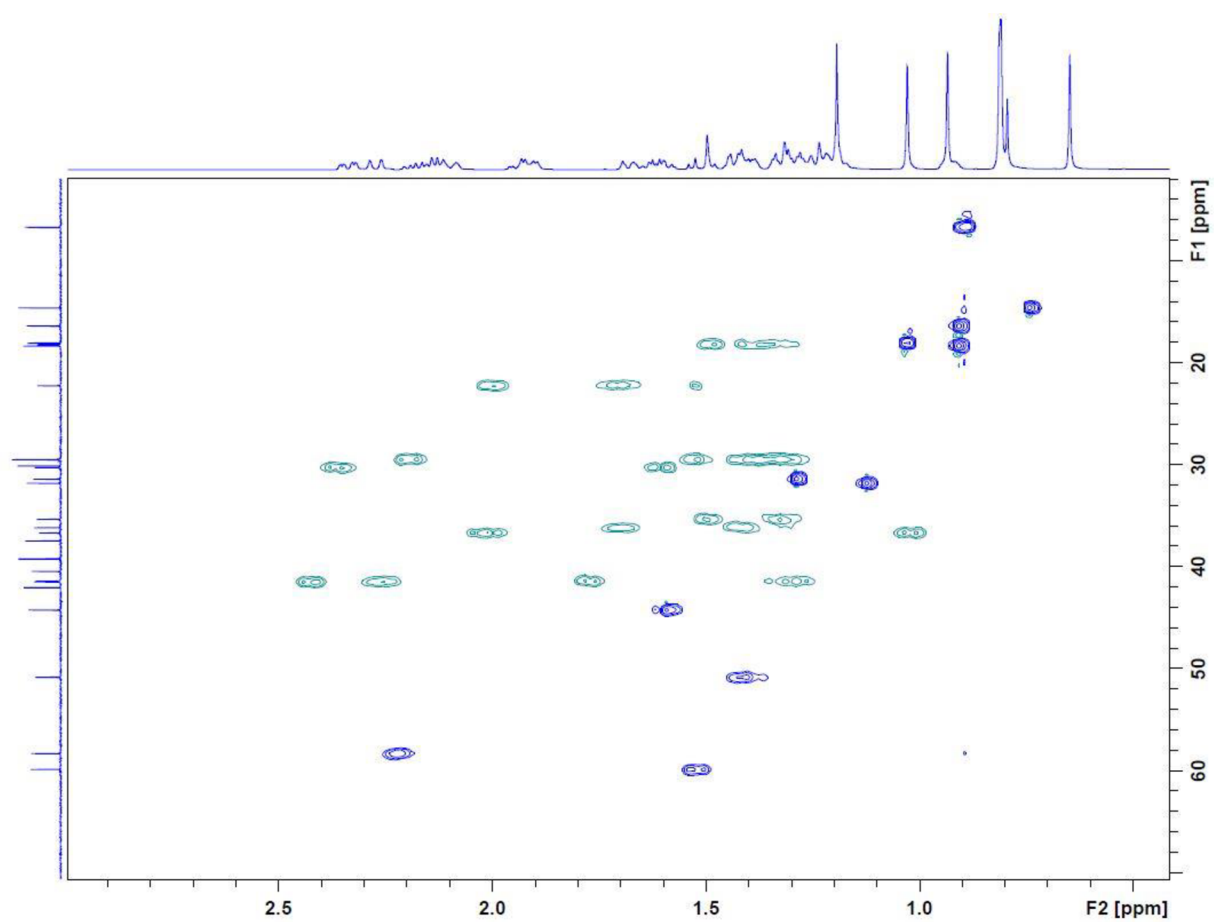

**S9 Fig.** HSQC spectrum of compound 2

Supplement: S9 Fig — (PDF) [file pone.0260956.s009.pdf]

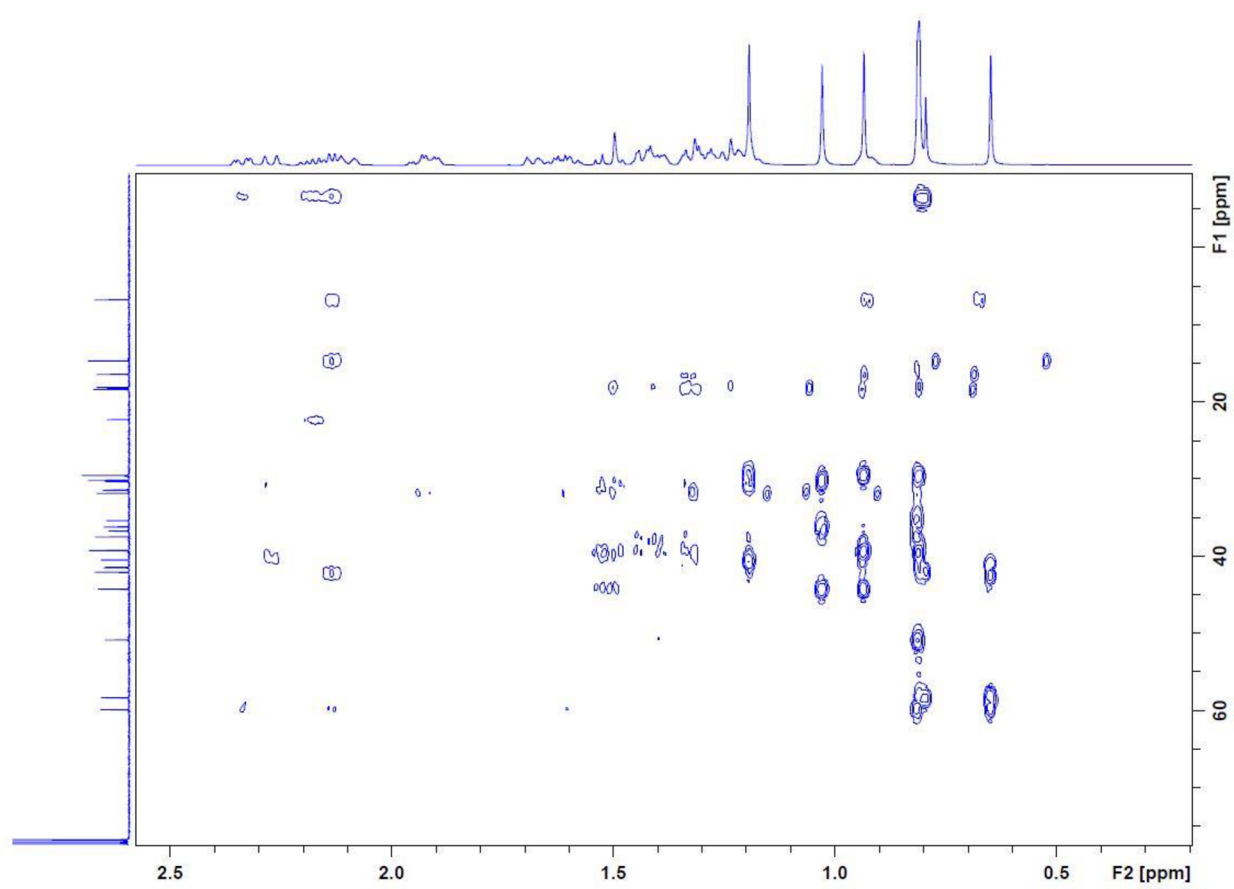

**S10 Fig. HMBC spectrum of compound 2**

Supplement: S10 Fig — (PDF) [file pone.0260956.s010.pdf]

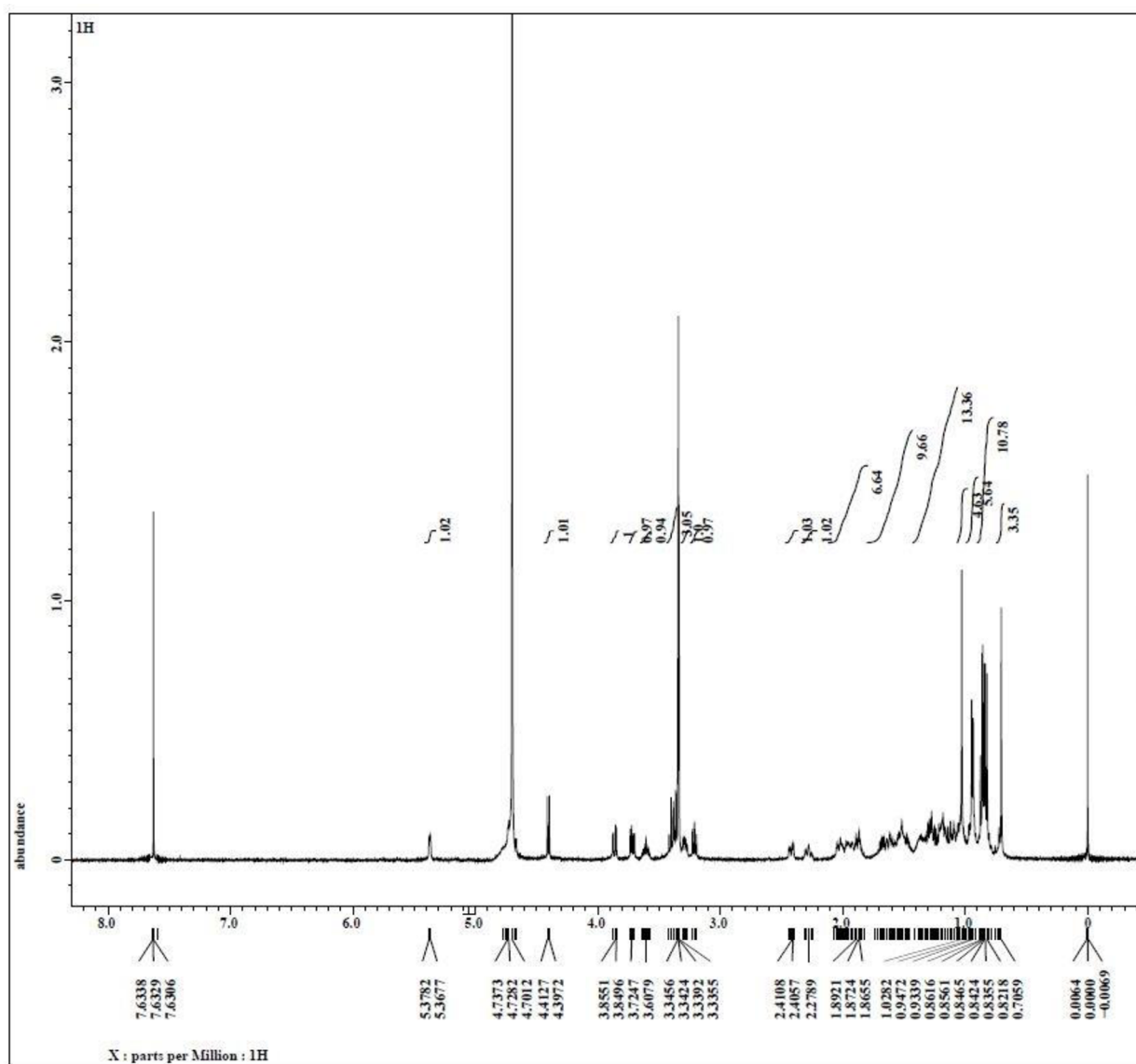

S11 Fig. <sup>1</sup>H NMR spectrum of compound 3

Supplement: S11 Fig — (PDF) [file pone.0260956.s011.pdf]

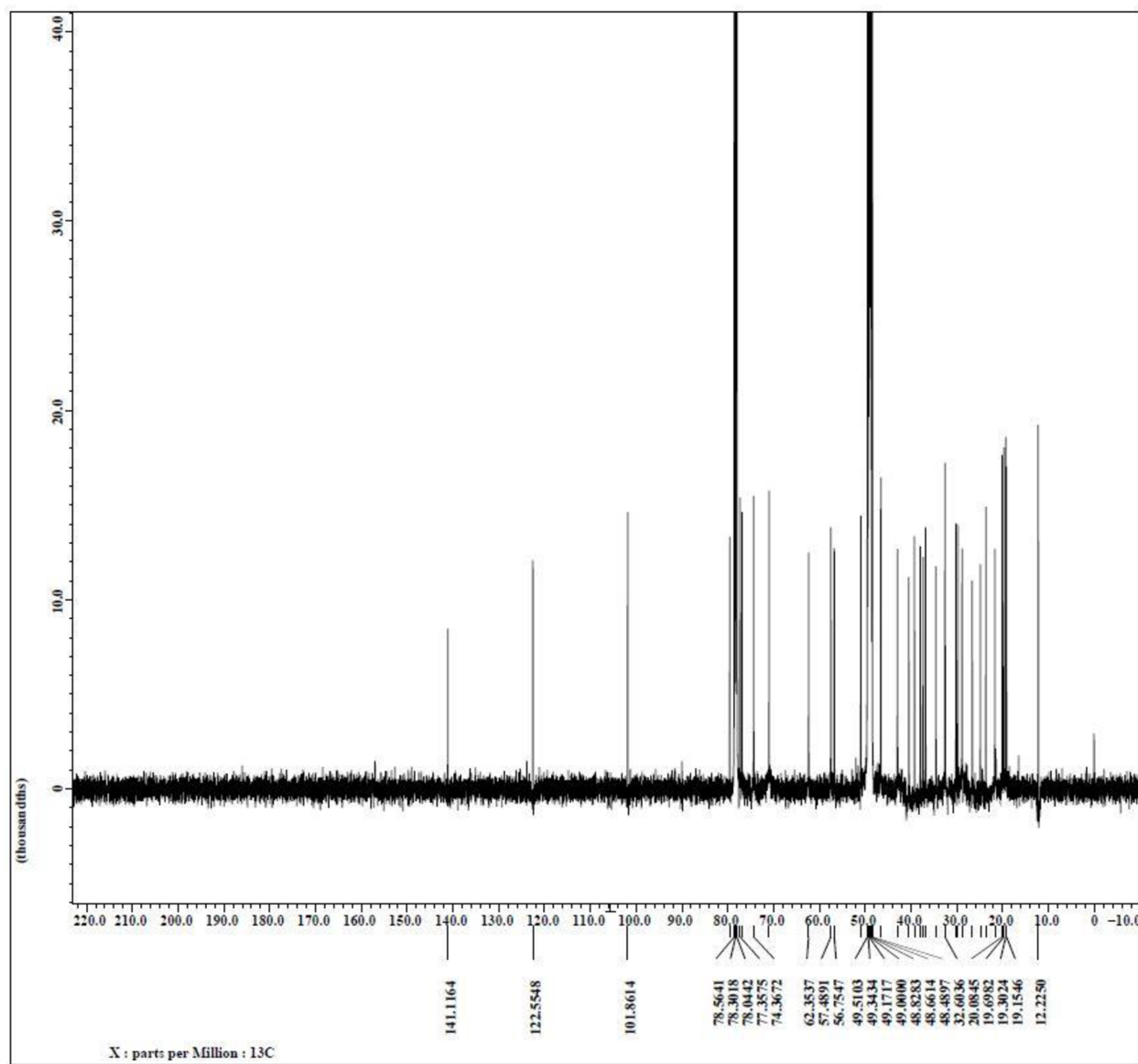

S12 Fig.  $^{13}\text{C}$  NMR spectrum of compound 3

Supplement: S12 Fig — (PDF) [file pone.0260956.s012.pdf]

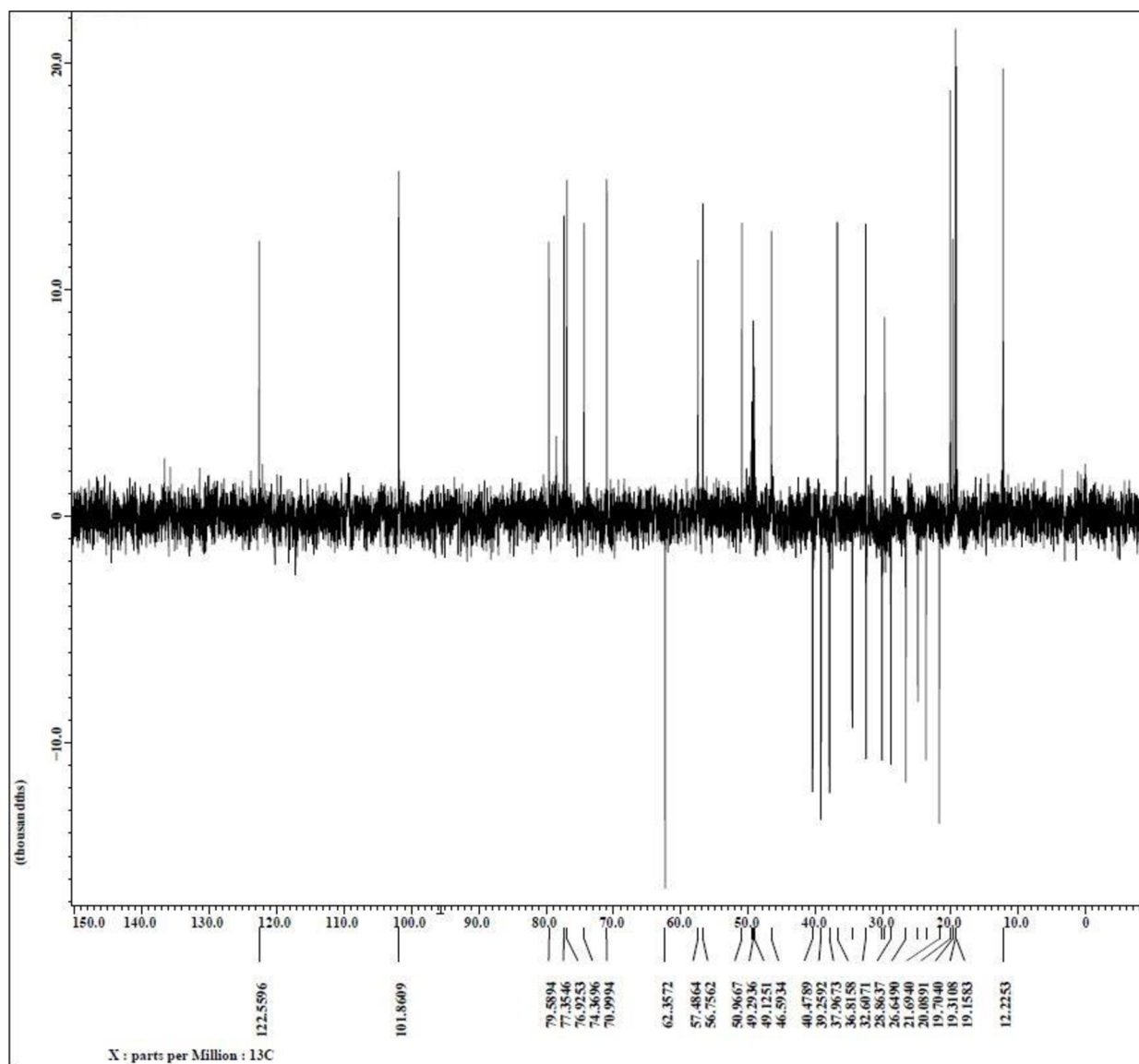

**S13 Fig. DEPT 135 spectrum of compound 3**

Supplement: S13 Fig — (PDF) [file pone.0260956.s013.pdf]

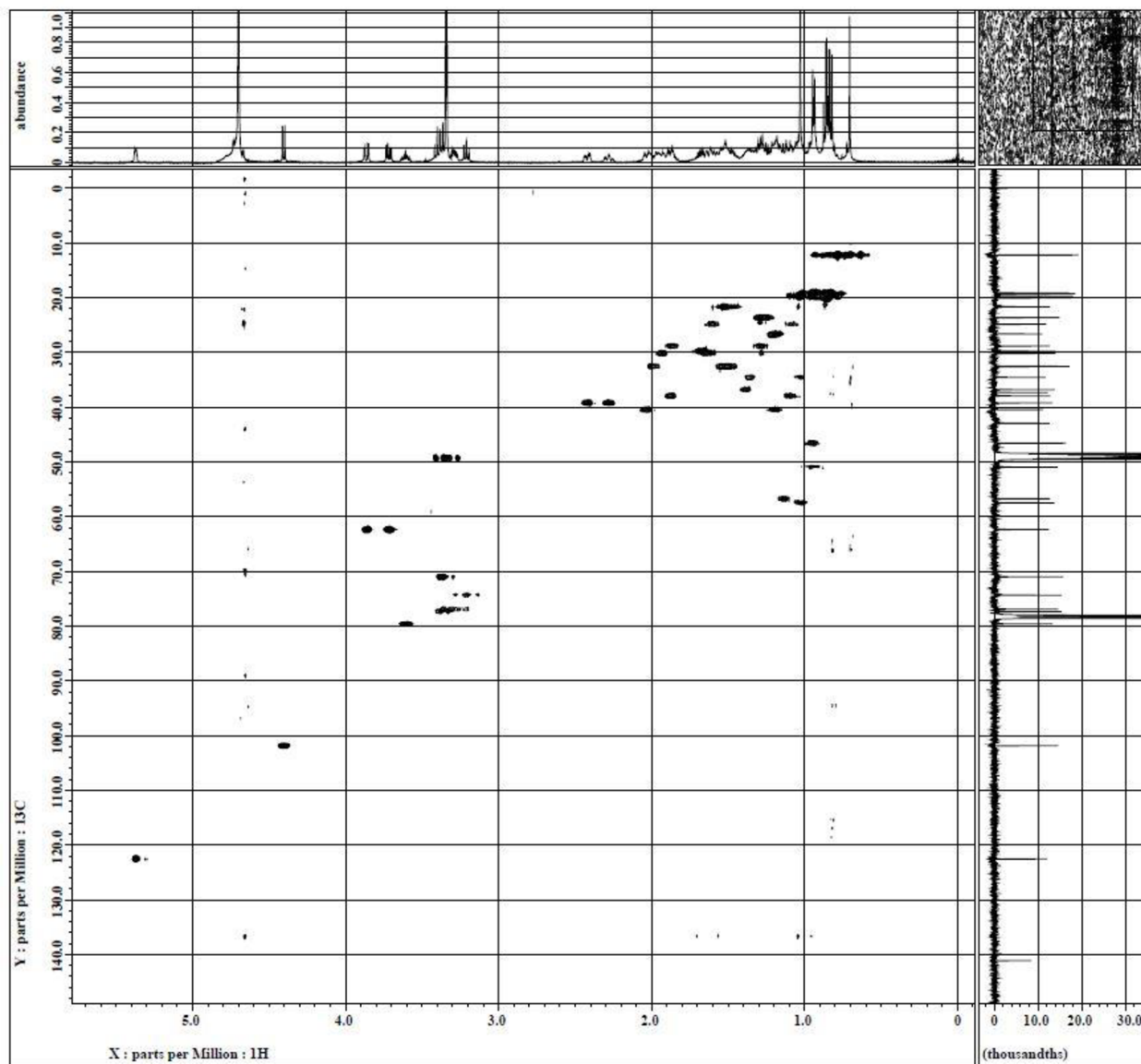

S14 Fig. HSQC spectrum of compound 3

Supplement: S14 Fig — (PDF) [file pone.0260956.s014.pdf]

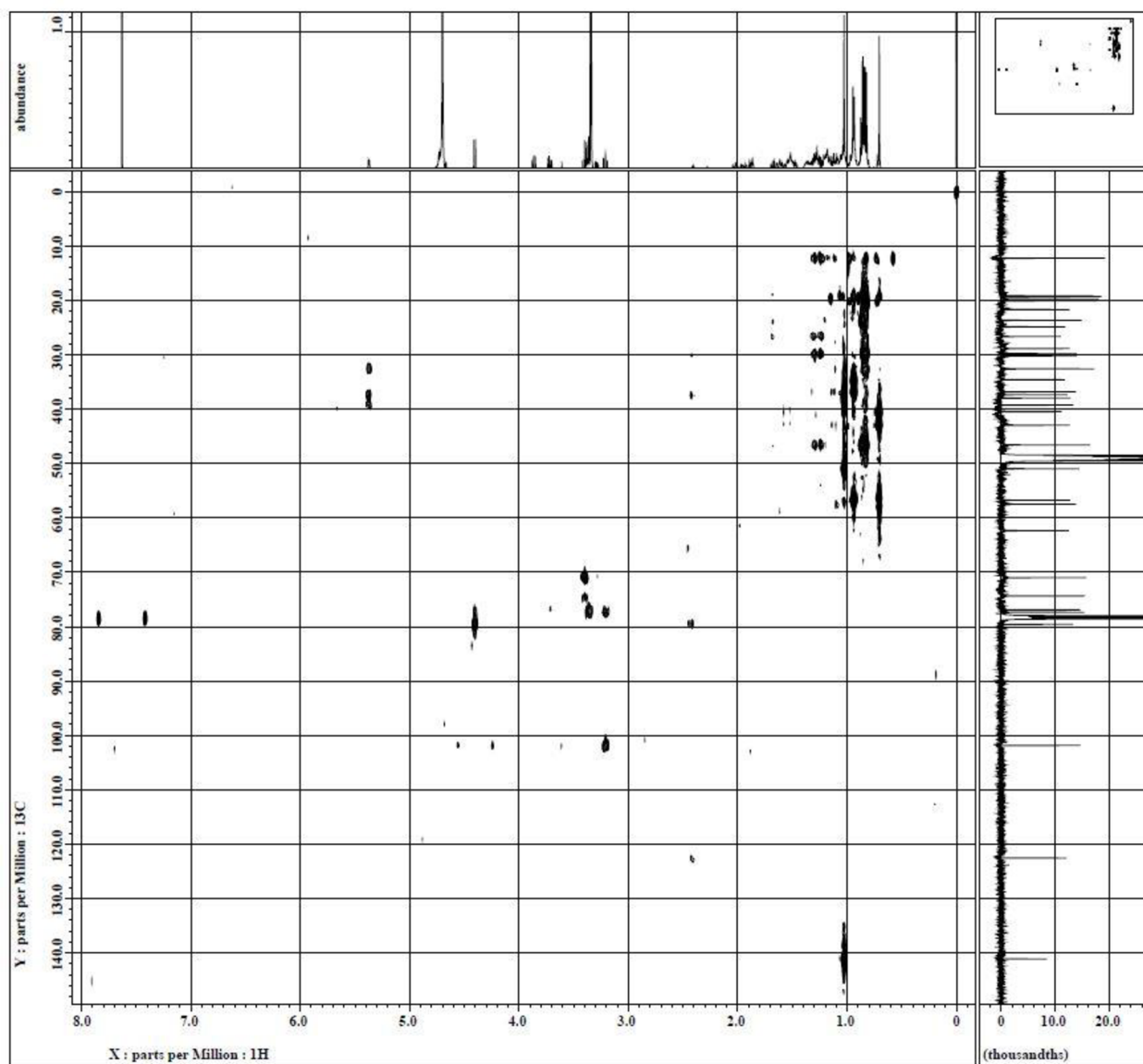

S15 Fig. HMBC spectrum of compound 3

Supplement: S15 Fig — (PDF) [file pone.0260956.s015.pdf]

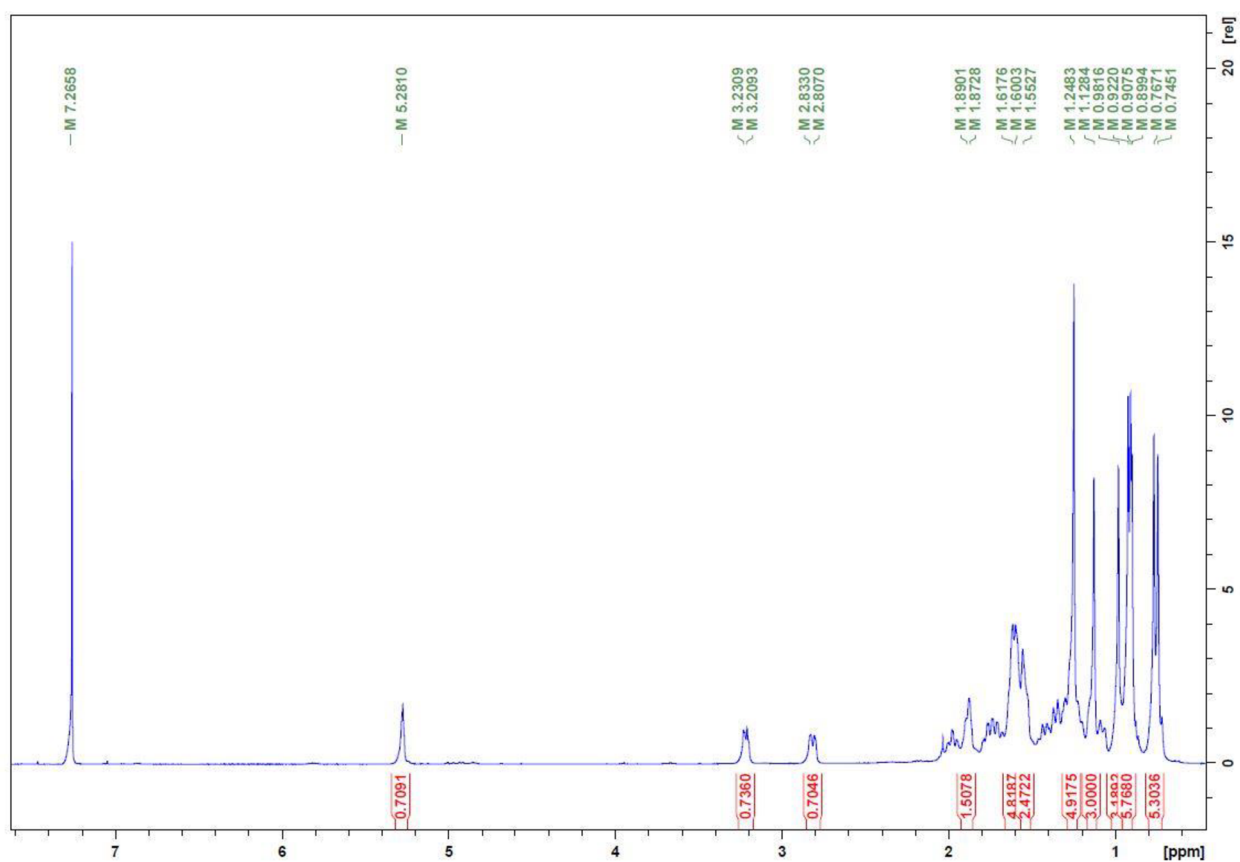

S16 Fig.  $^1\text{H}$  NMR spectrum of compound 4

Supplement: S16 Fig — (PDF) [file pone.0260956.s016.pdf]

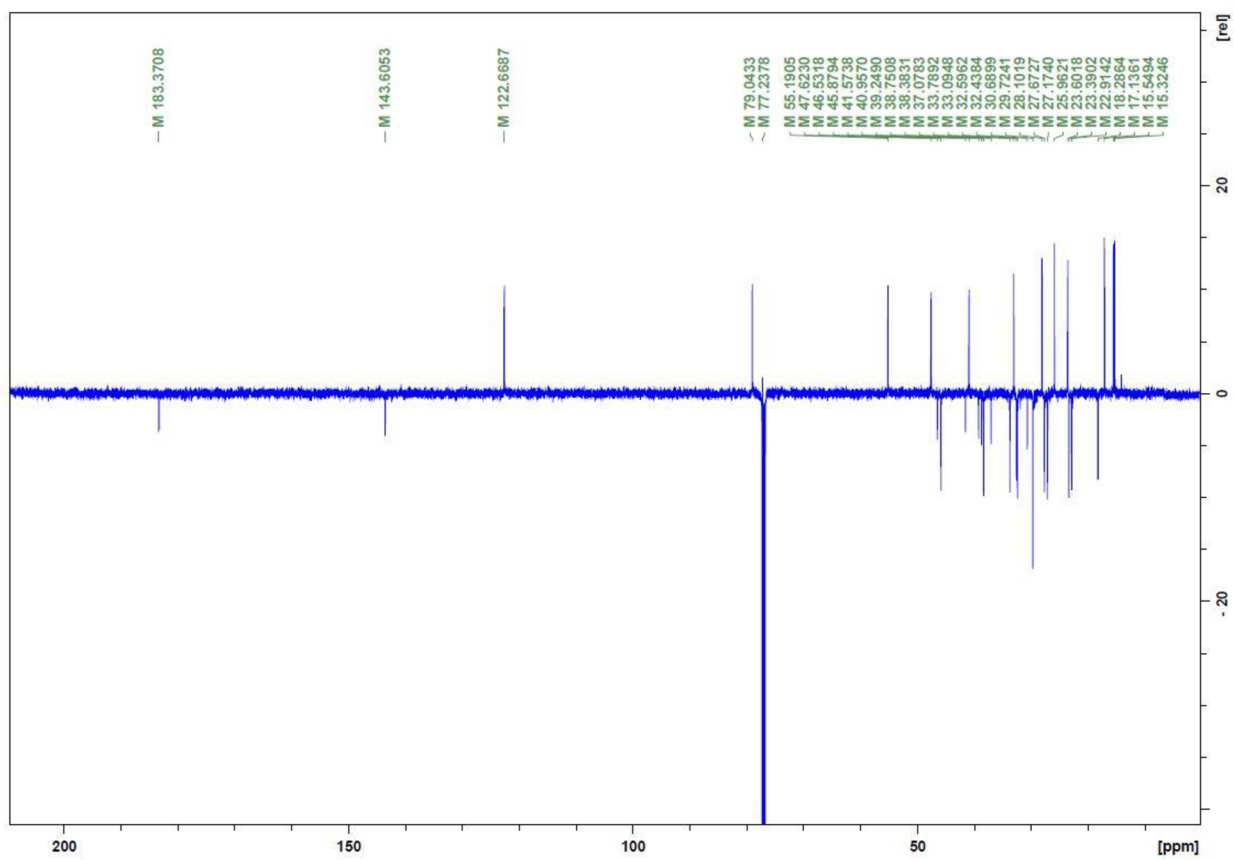

S17 Fig. DEPTQ spectrum of compound 4

Supplement: S17 Fig — (PDF) [file pone.0260956.s017.pdf]

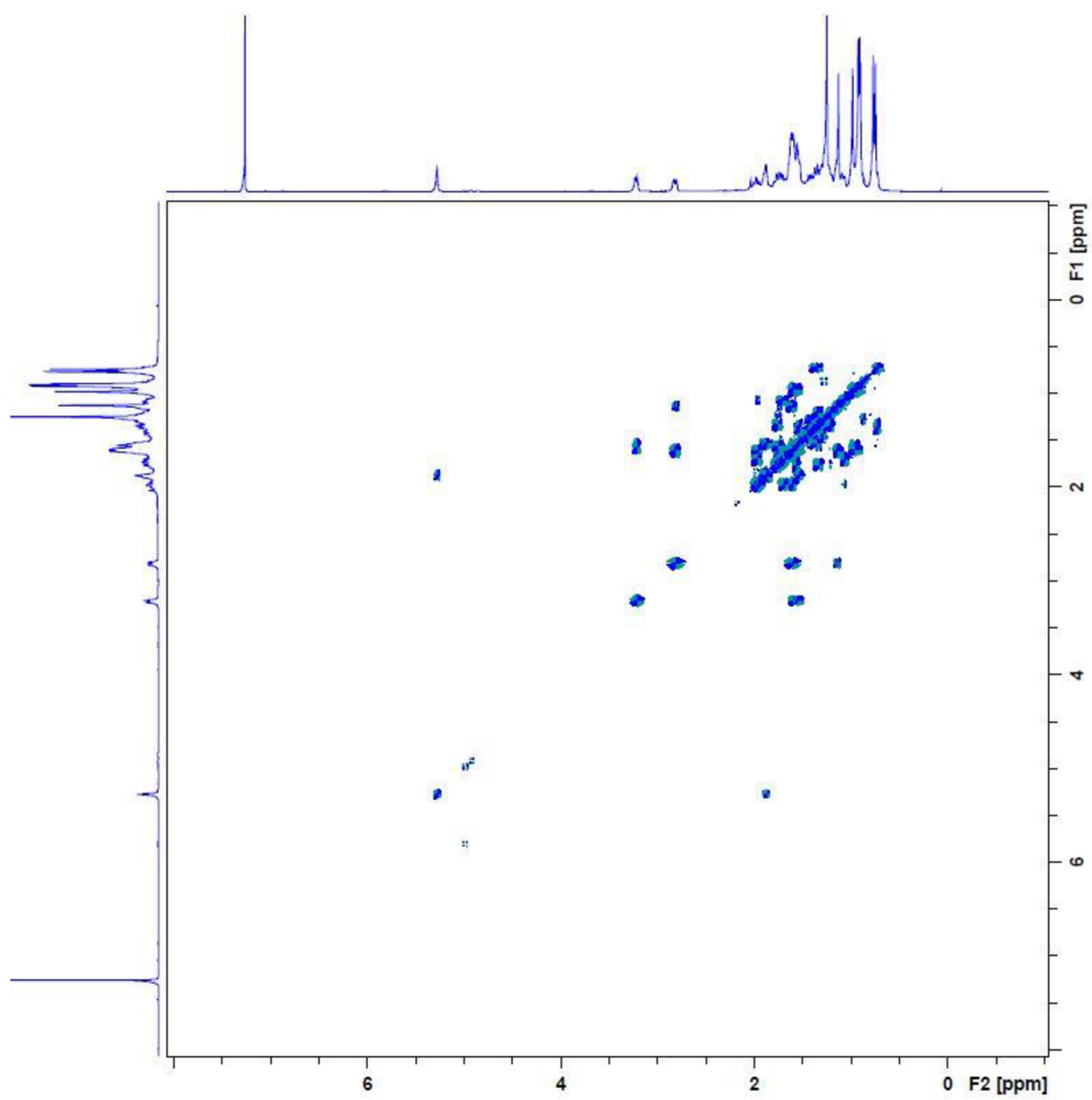

**S18 Fig. COSY spectrum of compound 4**

Supplement: S18 Fig — (PDF) [file pone.0260956.s018.pdf]

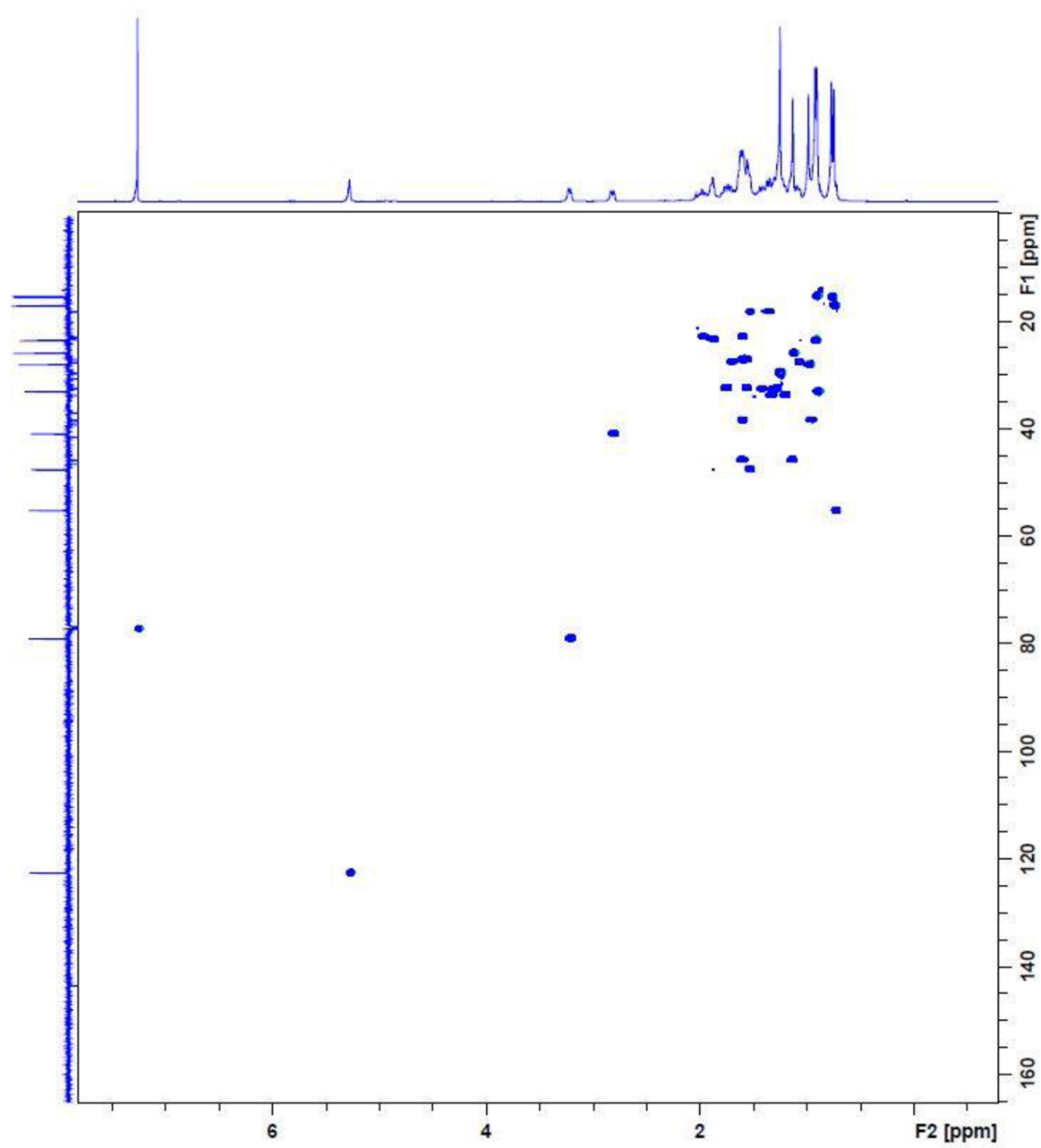

S19 Fig. HSQC spectrum of compound 4

Supplement: S19 Fig — (PDF) [file pone.0260956.s019.pdf]

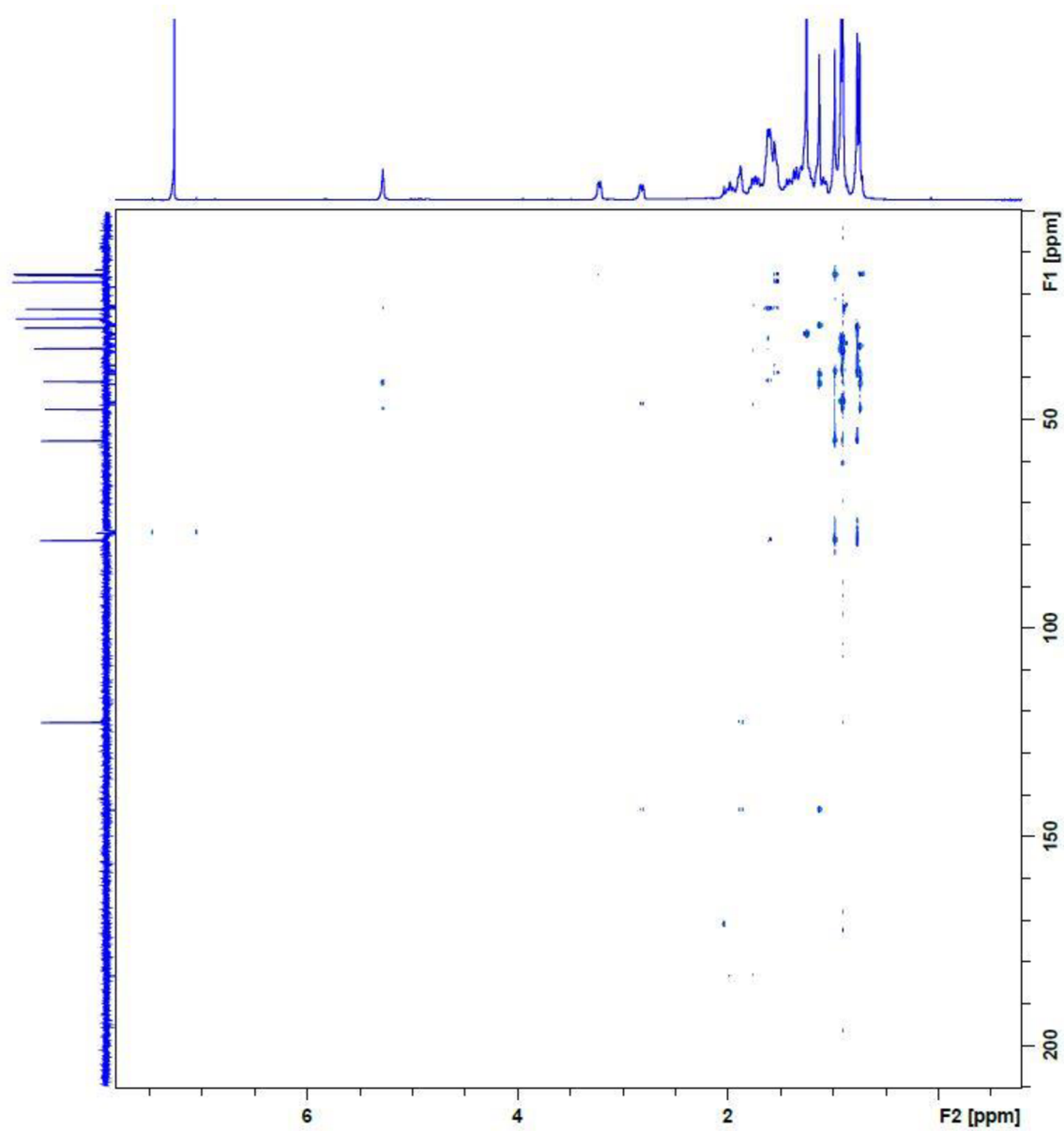

**S20 Fig. HMBC spectrum of compound 4**

Supplement: S20 Fig — (PDF) [file pone.0260956.s020.pdf]
