# Supplementary material for: Bioactive triterpenoids from Solanum torvum fruits with antifungal, resistance modulatory and anti-biofilm formation activities against fluconazole-resistant candida albicans strains
Source: PLoS One. 2021 Dec 28;16(12):e0260956. doi: 10.1371/journal.pone.0260956 (PMC8714089; doi:10.1371/journal.pone.0260956)
Supplement: S1 File — (PDF) [file pone.0260956.s021.pdf]

S1 Table: Effect of Betulinic acid (1) on the anti-*C. albicans* activity of fluconazole using the checkerboard assay

Organism	Betulinic acid (1)			Fluconazole		
	MIC alone	MIC combination	FIC	MIC alone	MIC combination	FIC
**CA-1**	0.032	0.016	0.5	0.064	0.008	0.125
**CA-2**	0.064	0.032	0.50	0.064	0.008	0.125
**CA-3**	0.064	0.064	1.0	0.064	0.008	0.125
**CA-4**	0.032	0.128	4.0	0.064	0.004	0.0625

All MICs are in mg/mL. Experiment was carried out in triplicate.

S2 Table: Effect of 3-oxo-friedelan-20α-oic acid (2) on the anti-*C. albicans* activity of fluconazole using the checkerboard assay

Organism	3-oxo-friedelan-20α-oic acid (2)			Fluconazole		
	MIC alone	MIC combination	FIC	MIC alone	MIC combination	FIC
**CA-1**	0.016	0.004	0.25	0.064	0.008	0.125
**CA-2**	0.016	0.016	1.0	0.064	0.008	0.125
**CA-3**	0.032	0.016	0.50	0.064	0.008	0.125
**CA-4**	0.032	0.064	2.0	0.064	0.008	0.125

All MICs are in mg/mL. Experiment was carried out in triplicate

S3 Table: Effect of Sitosterol-3-β-D-glucopyranoside (3) on the anti-*C. albicans* activity of fluconazole using the checkerboard assay

Organism	Sitosterol-3-β-D-glucopyranoside (3)			Fluconazole		
	MIC alone	MIC combination	FIC	MIC alone	MIC combination	FIC
**CA-1**	0.064	0.064	1.0	0.064	0.008	0.125
**CA-2**	0.064	0.064	1.0	0.064	0.008	0.125
**CA-3**	0.064	0.128	2.0	0.064	0.004	0.0625
**CA-4**	0.064	0.256	4.0	0.064	0.004	0.0625

All MICs are in mg/mL. Experiment was carried out in triplicate

S4 Table: Effect of Oleanolic acid (4) on the anti-*C. albicans* activity of fluconazole using the checkerboard assay

Organism	Oleanolic acid (4)			Fluconazole		
	MIC alone	MIC combination	FIC	MIC alone	MIC combination	FIC
**CA-1**	0.032	0.016	0.5	0.064	0.008	0.125
**CA-2**	0.064	0.064	1.0	0.064	0.008	0.125
**CA-3**	0.064	0.064	1.0	0.064	0.008	0.125
**CA-4**	0.064	0.128	2.0	0.064	0.008	0.125

All MICs are in mg/mL. Experiment was carried out in triplicate

S5 Table: Effect of Betulinic acid (1) on the anti-*C. albicans* activity of voriconazole using the checkerboard assay

Organism	Betulinic acid (1)			Voriconazole		
	MIC alone	MIC combination	FIC	MIC alone	MIC combination	FIC
**CA-1**	0.032	0.064	2.0	0.008	0.032	4.0
**CA-2**	0.064	0.016	0.25	0.016	0.064	4.0
**CA-3**	0.064	0.016	0.25	0.008	0.032	4.0
**CA-4**	0.032	0.032	1.0	0.004	0.016	4.0

All MICs are in mg/mL. Experiment was carried out in triplicate.

S6 Table: Effect of 3-oxo-friedelan-20α-oic acid (2) on the anti-*C. albicans* activity of voriconazole using the checkerboard assay

Organism	3-oxo-friedelan-20α-oic acid (2)			Voriconazole		
	MIC alone	MIC combination	FIC	MIC alone	MIC combination	FIC
**CA-1**	0.016	0.032	2.0	0.008	0.032	4.0
**CA-2**	0.016	0.016	1.0	0.016	0.016	1.0
**CA-3**	0.032	0.032	1.0	0.008	0.064	8.0
**CA-4**	0.032	0.032	1.0	0.004	0.032	8.0

All MICs are in mg/mL. Experiment was carried out in triplicate.

S7 Table: Effect of Sitosterol-3-β-D-glucopyranoside (3) on the anti-*C. albicans* activity of voriconazole using the checkerboard assay

Organism	Sitosterol-3-β-D-glucopyranoside (3)			Voriconazole		
	MIC $_{alone}$	MIC $_{combination}$	FIC	MIC $_{alone}$	MIC $_{combination}$	FIC
**CA-1**	0.064	0.128	2.0	0.008	0.048	6.0
**CA-2**	0.064	0.064	1.0	0.016	0.032	2.0
**CA-3**	0.064	0.016	0.25	0.008	0.032	4.0
**CA-4**	0.064	0.128	2.0	0.004	0.008	2.0

All MICs are in mg/mL. Experiment was carried out in triplicate

S8 Table: Effect of Oleanolic acid (4) on the anti-*C. albicans* activity of voriconazole using the checkerboard assay

Organism	Oleanolic acid (4)			Voriconazole		
	MIC $_{alone}$	MIC $_{combination}$	FIC	MIC $_{alone}$	MIC $_{combination}$	FIC
**CA-1**	0.032	0.032	1.0	0.008	0.032	4.0
**CA-2**	0.064	0.032	0.5	0.016	0.016	1.0
**CA-3**	0.064	0.032	0.5	0.008	0.016	2.0
**CA-4**	0.064	0.128	2.0	0.004	0.008	2.0

All MICs are in mg/mL. Experiment was carried out in triplicate

S9 Table: Anti-biofilm formation activity of Betulinic acid (1) against clinical fluconazole-resistant *C. albicans* strains

Concentration	Absorbance at 595 nm											
	CA-1			CA-2			CA-3			CA-4		
	1	2	3	1	2	3	1	2	3	1	2	3
**MIC**	0.286	0.281	0.289	0.240	0.237	0.244	0.318	0.314	0.322	0.261	0.234	0.263
**½ MIC**	0.355	0.344	0.365	0.338	0.326	0.350	0.391	0.378	0.405	0.349	0.338	0.360
**¼ MIC**	0.446	0.436	0.393	0.368	0.359	0.375	0.468	0.452	0.484	0.388	0.373	0.402
**$^1/_8$ MIC**	0.608	0.597	0.618	0.508	0.496	0.529	0.576	0.561	0.592	0.500	0.488	0.509

Blank determination: **0.874.** Each experiment was carried out in triplicate

S10 Table: Anti-biofilm formation activity of 3-oxo-friedelan-20α-oic acid (2) against clinical fluconazole-resistant *C. albicans* strains

Concentration	Absorbance at 595 nm											
	CA-1			CA-2			CA-3			CA-4		
	1	2	3	1	2	3	1	2	3	1	2	3
**MIC**	0.183	0.179	0.186	0.227	0.224	0.229	0.279	0.276	0.282	0.318	0.315	0.320
**½ MIC**	0.270	0.261	0.280	0.296	0.285	0.307	0.430	0.413	0.445	0.411	0.400	0.420
**¼ MIC**	0.323	0.308	0.338	0.366	0.355	0.376	0.452	0.437	0.466	0.542	0.526	0.556
**1/8 MIC**	0.502	0.392	0.410	0.437	0.422	0.450	0.564	0.552	0.575	0.635	0.624	0.648

Blank determination: **0.874.** Each experiment was carried out in triplicate

S11 Table: Anti-biofilm formation activity of Sitosterol-3-β-D-glucopyranoside (3) against clinical fluconazole-resistant *C. albicans* strains

Concentration	Absorbance at 595 nm											
	CA-1			CA-2			CA-3			CA-4		
	1	2	3	1	2	3	1	2	3	1	2	3
**MIC**	0.318	0.315	0.320	0.380	0.377	0.382	0.486	0.482	0.489	0.405	0.402	0.408
**½ MIC**	0.349	0.338	0.361	0.434	0.420	0.450	0.560	0.547	0.573	0.440	0.431	0.450
**¼ MIC**	0.442	0.430	0.453	0.537	0.528	0.546	0.619	0.608	0.631	0.553	0.542	0.565
**1/8 MIC**	0.618	0.608	0.627	0.630	0.613	0.640	0.685	0.675	0.695	0.658	0.729	0.670

Blank determination: **0.874.** Each experiment was carried out in triplicate

S12 Table: Anti-biofilm formation activity of Oleanolic acid (4) against clinical fluconazole-resistant *C. albicans* strains

Concentration	Absorbance at 595 nm											
	CA-1			CA-2			CA-3			CA-4		
	1	2	3	1	2	3	1	2	3	1	2	3
**MIC**	0.208	0.205	0.212	0.234	0.229	0.238	0.276	0.273	0.280	0.258	0.255	0.260
**½ MIC**	0.307	0.295	0.319	0.286	0.271	0.302	0.373	0.359	0.386	0.305	0.292	0.319
**¼ MIC**	0.385	0.375	0.394	0.366	0.356	0.375	0.497	0.480	0.513	0.371	0.360	0.319
**1/8 MIC**	0.413	0.399	0.428	0.429	0.411	0.447	0.578	0.565	0.592	0.488	0.472	0.506

Blank determination: **0.874.** Each experiment was carried out in triplicate
